# A Clinical Study of Anterior Abdominal Wall Hernias and Its Management: A Medical College Experience

**DOI:** 10.7759/cureus.67209

**Published:** 2024-08-19

**Authors:** Iqbal M Ali, Saurav Shetty K, Varun Shetty

**Affiliations:** 1 General Surgery, Dr. D. Y. Patil Medical College, Hospital and Research Centre, Dr. D. Y. Patil Vidyapeeth, Pune, IND; 2 General Surgery, Dr. D. Y. Patil Medical College, Hospital and Research Centre, Pune, IND

**Keywords:** postoperative pain, surgical site infection, seroma, incisional hernia, ventral hernia

## Abstract

Objective

We aim to evaluate the various risk factors contributing to the occurrence of anterior abdominal wall hernias and assess the various surgical modalities.

Materials and methods

This prospective observational research was conducted between 2022 and 2024 at a tertiary care health center, involving 100 participants with an anterior abdominal wall hernia diagnosis aged over 14 years. The study excluded patients under 14 years with bleeding diathesis, inherited coagulopathies, inguinal or femoral hernias, or recurrent ventral hernias. Participants underwent detailed clinical examinations and biochemical evaluations and underwent a primary ultrasonography (USG)/contrast-enhanced computed tomography (CECT) to determine defect size. Risk factors were documented, including age, gender, occupation, body mass index (BMI), comorbidities, previous surgery history, multiparity, smoking, chronic straining due to constipation or benign prostatic hyperplasia (BPH), malnutrition, chronic steroid use, chronic renal failure, and chronic liver disease. The surgical procedure was determined by the same surgical team for all cases. Standard antibiotic prophylaxis and preoperative painting/draping protocols were followed in all cases. Intraoperatively, intraoperative time (in hours) was documented. Postoperative parameters included pain, hematoma formation, seroma formation, surgical site infections (SSIs), and early recurrence. These intra- and postoperative findings constituted the primary outcome parameters. Secondary outcome parameters included hospital stay duration and time taken to return to work. Data analysis was performed using Statistical Package for the Social Sciences (SPSS) version 16 (IBM SPSS Statistics, Armonk, NY) software.

Results

The study analyzed the occurrence of ventral hernias in 100 patients, with the fourth decade having the highest occurrence (n=42 (42%)). The majority of the participants were male (female: n=47 (47%), male: n=53 (53%)). The majority of the participants were laborers, and 24% (n=24) were office workers. The study found that hypertension, diabetes mellitus, and chronic obstructive pulmonary disease were risk factors for hernias. Other risk factors included obesity, previous history of surgery, multiparity, smoking, chronic straining, malnutrition, and chronic steroid use. The most common type of ventral hernia was umbilical hernia (n=33 (33%)), followed by paraumbilical hernias (n=30 (30%)), and incisional hernias (n=20 (20%)). Of the 100 patients, 74% underwent open hernia repair, with the mean operation time being minimal in cases managed with laparoscopic repair (2.5±0.67 hours). Postoperative pain was highest with the Rives-Stoppa (RS) repair with component separation group. The incidence of surgical site infection was the maximum among cases of open anatomical repair (41.7%), followed by RS repair (31.3%), while it was the minimum in laparoscopic repair (3.7%). Early recurrence was lower in the laparoscopic group (n=1 (3.7%)).

Conclusion

The study highlights risk factors for abdominal wall hernia and management approaches. Understanding these is crucial for identifying and preventing recurrence. Surgeons must choose the right surgical approach based on patient health and symptoms to achieve desired outcomes and minimize complications. In addition, surgical expertise, availability of resources, and knowledge of what works best for the surgeon constitute important determinants of surgical outcomes.

## Introduction

Sir Astley Paston Cooper had asserted that "no disease of the human body belonging to the province of the surgeon requires in its treatment a better combination of accurate anatomical knowledge with surgical skill than hernia in all its varieties" [1].

An anterior abdominal wall hernia, also referred to as a ventral hernia, is a condition in which intra-abdominal or pre-peritoneal contents are eviscerated through a normal/abnormal fascial opening in the abdominal wall. This condition may or may not lead to a loss of abdominal domain and/or abdominal visceral disproportion. We can classify ventral hernias as either spontaneous (primary), acquired (secondary), or based on their location on the abdominal wall. Ventral hernia includes epigastric, umbilical/paraumbilical, and incisional hernia [2].

The primary obstacles in hernia management are the determination of the appropriate surgical method, including laparoscopic or open surgery, anatomical or mesh repair, the specific type of mesh to use, and the layer of mesh placement. Sublay (between the rectus muscle and posterior rectus sheath) meshplasty has been shown to be associated with a lower incidence of seroma formation. Additionally, it is imperative to ascertain the most effective mesh placement to minimize the risk of recurrence and guarantee the most durable repair [3].

Achieving a tension-free repair during surgery is a challenging endeavor. Primary closure using either a figure of 8 or double breasting techniques was the initial method for repairing a ventral hernia. One example of the more sophisticated closure techniques that have emerged over the years is component separation and repair with tension-free, sublay meshplasty. In hernia surgery, the tension-free repair is a game changer, among other groundbreaking ideas. Inguinal and incisional hernias are less likely to recur due to mesh prostheses, which approximate the fascial defect. LeBlanc and Booth described the initial laparoscopic incisional hernia repair in 1993 [4].

Modern surgical practices prioritize aesthetics while simultaneously striving to reduce postoperative morbidity and hospital stay duration. Laparoscopic surgery has become increasingly significant due to its minimally invasive approach, shorter hospital stay, and enhanced cosmesis. Over the years, laparoscopic surgery has gained popularity, owing to its shorter recovery period, lower postoperative pain, and earlier return to daily activities. Additionally, defect size and availability of surgical expertise/resources also play an important role in decision-making, pertaining to the type of surgery [5]. Several comparative studies, such as those by Wassenaar et al. [6] and Bencini et al. [7], have shown that the laparoscopic method leads to a faster recovery to normal activity levels, shorter hospitalization, and less surgical discomfort.

The purpose of this study was to investigate the various risk factors associated with the development of abdominal wall hernias and underscore the extensive array of surgical techniques employed to address them.

## Materials and methods

This prospective observational research was conducted between 2022 and 2024 at a tertiary care health center. Included in the investigation were 100 consenting study participants admitted at our institution with a diagnosis of anterior abdominal wall hernia aged greater than 14 years. Institutional ethics clearance was obtained prior to beginning the study from Dr. D. Y. Patil Vidyapeeth Institutional Ethics Sub-committee (IESC/338/2022). The study was initiated only after obtaining informed consent from all the participants. The study excluded patients under the age of 14 years who had bleeding diathesis or inherited coagulopathies. Patients with inguinal or femoral hernias were also excluded from the trial. Because a broader spectrum of factors influence the occurrence of recurrent ventral hernias, they were omitted from the study.

All the included participants were evaluated thoroughly by means of detailed clinical examination and biochemical evaluation (including complete blood count, serum electrolytes, renal and liver function tests, and coagulation profile), and the findings were logged in dedicated proformas created for this research. The patients underwent a primary ultrasonography (USG)/contrast-enhanced computed tomography (CECT) of the abdomen and pelvis on admission in order to ascertain the contents and delineate the defect size. The demographic profile and associated risk factors were documented. These risk factors included age, gender, occupation, body mass index (BMI), comorbidities (diabetes mellitus (DM), hypertension (HTN), and chronic obstructive pulmonary disease (COPD)), previous history of surgery, multiparity, smoking, chronic straining due to constipation or benign prostatic hyperplasia (BPH), malnutrition, chronic steroid use, chronic renal failure, and chronic liver disease.

All patients were then subjected to the surgical procedure depending upon the defect size and general condition of the patient. The decision on the type of procedure was made by the same surgical team for all cases. The surgical procedures offered included open (anatomical repair, Rives-Stoppa (RS) repair, and component separation) and laparoscopic (extended totally extraperitoneal repair (e-TEP) and intraperitoneal onlay meshplasty (IPOM/IPOM plus)) procedures.

All patients received a dose of an injectable broad-spectrum antibiotic half an hour prior to incision (based on the institute antibiotic policy). Every patient is scrubbed using 7% povidone-iodine prior to shifting to the operating theater. In addition, a 5% povidone-iodine solution is used for painting the surgical field prior to surgery. No specific surgical site infection (SSI) bundles were employed while conducting this study. The intraoperative time for each procedure was documented for later comparison. Postoperatively, the parameters recorded included postoperative pain (according to the visual analog scale (VAS)), postoperative seroma formation, hematoma formation, surgical site infections (SSI), and early recurrence. Secondary outcome parameters included the duration of hospital stay (in days) and time to get back to work (in days). Postoperatively, the patients were followed up to six months to look for early recurrence.

Descriptive statistics were computed to delineate the study sample. After the completion of data collection, data analysis was achieved using Statistical Package for the Social Sciences (SPSS) version 16 software (IBM SPSS Statistics, Armonk, NY). Qualitative data was represented in the form of frequency and percentage. The association between qualitative variables was assessed using the chi-square test. Analysis of quantitative data between the two groups was done using an unpaired t-test if the data passed the normality test and the Mann-Whitney test if the data failed the normality test. A p-value < 0.05 was taken as the level of significance.

## Results

In our study, the age group with the highest occurrence of ventral hernias was the fourth decade, i.e., 42% (n=42) (Table 1). The study participants' average age was 46.84±14.83 years (mean±standard deviation (SD)). Of the 100 patients, 47% (n=47) were females, while 53% (n=53) were males. The observed sex ratio was 1.13:1, indicating a slight male preponderance (Table 2). According to the modified Kuppuswami scale, the majority of the study participants fell within the lower middle (n=40 (40%)) and lower socioeconomic strata (n=20 (20%)) (Table 3). Laborers constituted 42% (n=42) of the participants, while office workers made up the second largest occupational group with 24% (n=24) (Table 4).

**Table 1:** Age-wise distribution of the study participants (N=100)

Age group (years)	Number	%
≤20	1	1%
21-40	39	39%
41-60	42	42%
>60	18	18%
Total	100	100%

**Table 2:** Gender-wise distribution of the study participants (N=100)

Gender	Number	%
Female	47	47%
Male	53	53%
Total	100	100%

**Table 3:** Distribution of patients based on socioeconomic status (using the modified Kuppuswami scale) The modified Kuppuswami scale is a scale utilizing the following parameters to estimate the socioeconomic status of patients: (1) the educational status of the head of the family, (2) the occupation of the head of the family, and (3) the monthly income of the family. Based on these parameters, a cumulative score is allotted, and the socioeconomic status is estimated.

Socioeconomic status	Number	%
Upper class	17	17%
Upper middle class	17	17%
Lower middle class	40	40%
Upper lower class	6	6%
Lower class	20	20%
Total	100	100%

**Table 4:** Distribution of study participants based on occupation

Occupation	Number	%
Laborer/daily wager	42	42%
Office worker	24	24%
Farmer	18	18%
Education system	6	6%
Unemployed	10	10%
Total	100	100%

The majority of our study participants, i.e., 37% (n=37), presented with HTN, followed by 27% (n=27) with DM, and 11% (n=11) with COPD, all of which are considered contributory risk factors for the occurrence of hernia. The other risk factors encountered included obesity (n=25 (25%)), previous history of surgery (n=30 (30%)), multiparity (n=15 (15%)), smoking (n=19 (19%)), and chronic straining (n=24 (24%)), either secondary to chronic constipation (n=16 (16%)) or BPH (n = 8 (8%)). People who had surgery in the past and developed incisional hernias were mostly those who had an open appendectomy (43.3%) or a lower segment cesarean section (LSCS) delivery (n=12 (40%)). In addition, long-standing conditions such as chronic liver disease (n=1 (1%)), chronic steroid use (n=2 (2%)), and malnutrition (n=1 (1%)) also contributed to the occurrence of anterior abdominal wall hernia (Table 5).

**Table 5:** Frequency of risk factors predisposing to the occurrence of anterior abdominal wall hernia HTN: hypertension, DM: diabetes mellitus, COPD: chronic obstructive pulmonary disease, LSCS: lower segment cesarean section, BPH: benign prostatic hyperplasia

Risk factors		Number	%
Comorbidities (n=75)	HTN	37	37%
	DM	27	27%
	COPD	11	11%
Previous surgery (n=30)	Open appendectomy	13	13%
	LSCS	12	40%
	Exploratory laparotomy	4	13.3%
	Hysterectomy	1	3%
Obesity		25	25%
Smoking/tobacco		19	20%
Chronic straining (n=24)	Chronic constipation	16	16%
	BPH	8	8%
Multiparity		15	15%
Malnutrition		1	1%
Chronic steroid use		2	2%
Chronic diseases	Chronic renal failure	0	0%
	Chronic liver disease	1	1%

Among the 100 patients, the most frequent type of ventral hernia encountered was umbilical hernia (n=33 (33%)), followed by paraumbilical hernias (n=30 (30%)) and incisional hernias (n=20 (20%)) (Table 6). The most common clinical presentation of hernia in our study sample was pain (n=36 (36%)), followed by incidental diagnosis during a hospital visit (n=24 (24%)). Among the study participants, 21% (n=21) had an obstructed hernia, 19% (n=19) had strangulated hernia, and 60% (n=60) had uncomplicated hernias.

**Table 6:** Different types of abdominal wall hernias encountered in our study

Type of hernia	Number	%
Umbilical	33	33%
Paraumbilical	30	30%
Incisional	20	20%
Epigastric	13	13%
Spigelian	2	2%
Lumbar	1	1%
Subcostal	1	1%
Total	100	100%

Out of the 100 patients, 74% (n=74) underwent open hernia repair, while 26% (n=26) underwent laparoscopic surgery. The majority of the patients had a defect size of <4 cm (n=51 (51%)), whereas 20% (n=20) had defects larger than 10 cm. The patients underwent the surgical procedure (open/laparoscopic) (Table 7). Due to hernia-related complications (obstruction or strangulation), 40% (n=40) of the patients underwent emergency surgery, while 60% (n=60) underwent elective surgery.

**Table 7:** Distribution of patients according to the surgical procedure performed RS: Rives-Stoppa, e-TEP: extended totally extraperitoneal repair, IPOM: intraperitoneal onlay meshplasty

Type of surgery	Number	%
Open surgery		
Anatomical repair	24	24%
RS repair	32	32%
RS repair + component separation	17	17%
Laparoscopic repair		
e-TEP	17	17%
IPOM/IPOM plus	10	10%
Total	100	100%

The mean operation time was minimal in cases managed with laparoscopic repair (2.59±0.67 hours), while it was highest for cases of open RS repair with component separation (3.97±1.03 hours) (Table 8). Student's t-test demonstrated a statistically significant association between the type of surgical procedure and the intraoperative time (p=0.01; p<0.05).

**Table 8:** Association of type of surgery with intraoperative time (hours) The statistical analysis included an independent t-test to assess the association between the two variables. The p-value for this test was 0.012±0.001, which was less than the level of significance of 0.05. This indicates that there was a statistically significant difference in the operative timings of both types of procedures. SD: standard deviation, RS: Rives-Stoppa, e-TEP: extended totally extraperitoneal repair, IPOM: intraperitoneal onlay meshplasty

Type of surgery	Number	Intraoperative time (hours)	
		Mean	SD
Open surgery			
Anatomical repair	24	3.20	1.22
RS repair	32	2.62	0.85
RS repair + component separation	17	3.97	1.03
Laparoscopic surgery			
Laparoscopic repair (e-TEP/IPOM/IPOM plus)	27	2.59	0.67
p-value = 0.012±0.001			

Along the same lines, postoperative pain and discomfort, as measured by the VAS, were highest in the open RS repair with component separation group (p=0.02; p<0.05). The open surgical group recorded the highest incidence of postoperative seroma formation (n=6 (8.21%)), especially with open anatomical repair with onlay mesh placement, and a statistically significant difference was noted between the different management groups (p=0.025; p<0.05) (Table 9).

**Table 9:** Association of the type of hernia surgery with the occurrence of postoperative seroma formation The statistical analysis included an independent t-test to assess the association between the two variables. The p-value for this test was 0.025±0.002, which was less than the level of significance of 0.05. This indicates that there was a statistically significant difference in the postoperative seroma formation rates. RS: Rives-Stoppa, e-TEP: extended totally extraperitoneal repair, IPOM: intraperitoneal onlay meshplasty

Type of surgery	Number	Postoperative seroma	%
Open surgery			
Anatomical repair	24	3	12.5%
RS repair	32	2	6.25%
RS repair + component separation	17	1	5.88%
Laparoscopic surgery			
Laparoscopic repair (e-TEP/IPOM/IPOM plus)	27	1	3.7%
p-value = 0.025±0.002			

The incidence of surgical site infection was highest among cases managed by open anatomical repair (41.7%), followed by RS repair (31.3%), while it was lowest in the laparoscopic repair group (3.7%). Most of the surgical site infection cases were superficial in nature; however, two cases in the anatomical repair group (8.3%) and one case in the RS plus component separation group (5.9%) had organ/space SSI.

Similarly, patients who underwent open procedures took longer to return to work, ranging from 12.12 to 4.58 days, compared to those who underwent laparoscopic procedures, which took 5.02 to 1.73 days, with a statistically significant difference (p=0.012). A higher incidence of early recurrence (<6 months post-surgery) was observed among patients with the open repair (n=3 (11.46%)) as compared to the laparoscopic group (n=1 (3.7%)). However, the comparison was not statistically significant (p=0.435) (Table 10).

**Table 10:** Association of the type of surgery with the incidence of early recurrence The statistical analysis included an independent t-test to assess the association between the two variables. The p-value for this test was 0.435±0.032, which was greater than the level of significance of 0.05. This indicates that there was no statistically significant difference in the early recurrence rates of both types of procedures. RS: Rives-Stoppa, e-TEP: extended totally extraperitoneal repair, IPOM: intraperitoneal onlay meshplasty

Type of surgery	Number	Recurrence	%
Open surgery			
Anatomical repair	24	2	8.33%
RS repair	32	1	3.13%
RS repair + component separation	17	0	0%
Laparoscopic surgery			
Laparoscopic repair (e-TEP/IPOM/IPOM plus)	27	1	3.7%
p-value = 0.435±0.032			

## Discussion

Surgeons worldwide encounter a substantial surgical challenge in terms of abdominal wall hernias. Incisional hernias are the most prevalent, with estimates ranging from 2% to 20% [7,8]. The global incidence of ventral hernias ranges from 20% to 50%. The current study provided a comprehensive overview of the various types of ventral hernias, their associated risk factors, and the available surgical treatment alternatives. The study also examined the postoperative complications that transpired during the patient's hospitalization.

Similar to the results of the studies by Jin et al. [9] and Pandya et al. [10], the mean age of the participants in our study was 46.84±14.83 years. Purushotham et al. [11] conducted a study where participants in the laparoscopy group averaged 32.38 years, while those in the open group averaged 47.81 years. In our investigation, the male population made up 53% of the total patients. A higher proportion of male participants was also present in previous studies, including those conducted by Korukonda et al. [12].

Our analysis identified several prominent risk factors. These comprised a prior history of surgery (n=30 (30%)), obesity (n=25 (25%)), multiple childbirths (n=15 (15%)), and smoking (n=19 (19%)) as shown in Table 5. Hariyani et al. [13] identified obesity as the primary risk factor for the condition, accounting for 36% of cases. The second most prevalent risk factor was constipation, which accounted for 26% of cases. Comorbidities, particularly HTN, accounted for 22% of cases. The study identified four patients (8%) as having multiparity. Jadhav et al. [14] in their study revealed that 34% of the patients were obese, 24% experienced chronic constipation, and 14% had a history of heavy lifting.

Often observed in the immediate postoperative period, incisional hernias can also develop up to a decade following surgery. These late-onset hernias may be the result of minor hernias that were previously undetected. Hariyani et al. [13] discovered that 48% of hernias were the result of gynecological surgeries, with hysterectomy accounting for 22% and LSCS contributing 8%. Exploratory laparotomy (38%), open appendectomy (8%), and laparoscopy port site hernia repair (4%) were the most frequently performed surgeries. According to the research by Jadhav et al. [14], gynecological treatments account for 55.8% of hernia cases. Similarly, our data indicated that the most frequently performed operation was an open appendectomy, which accounted for 13% of cases. The prior LSCS was the second most frequently performed operation, with 12% of cases.

Umbilical hernias were the most prevalent form of abdominal wall hernias in the current investigation, accounting for 33% of cases. Paraumbilical hernias followed at 30%. Mukadam et al. [15] conducted a study on a cohort of 60 patients with ventral hernias, identifying umbilical hernia as the most prevalent type, accounting for 60% of all cases. Incisional hernia is the most prevalent subtype of ventral hernia, comprising 54% of cases, according to Hariyani et al. [13].

The study documented intraoperative time, postoperative discomfort, postoperative seroma formation, SSI rate, time to return to work, and early recurrence (Table 11). Basheer et al. [16], in their study, showed that the laparoscopic repair procedure, lasting 86 minutes, was significantly shorter than the open repair procedure, which lasted 91 minutes. Furthermore, Lomanto et al. [17] found that laparoscopic ventral hernia repair reduced the surgical procedure's duration. The operative time between the two groups did not exhibit any significant variation, as determined by Mohamed et al. [18].

**Table 11:** Outcome parameters measured while comparing surgical methods utilized in the management of anterior abdominal wall hernias VAS: visual analog scale, SSI: surgical site infection

Parameter/complications	Open (n=73)	Laparoscopic (n=27)	p-value
Primary outcome measures			
Postoperative pain (as per VAS)	5.15±1.05	3.27±0.23	0.002±0.001
Postoperative seroma formation	6 (8.21%)	1 (3.7%)	0.161±0.002
Postoperative SSI	22 (30.13%)	1 (3.7%)	0.04±0.01
Early recurrence (within 6 months)	3 (4.1%)	1 (3.7%)	0.435±0.003
Secondary outcome measures			
Duration of hospital stay (days)	4.04±0.05 days	2.74±0.03 days	0.543±0.02
Time to get back to work (days)	12.21±0.04 days	5.0±1.73 days	0.012±0.001

Purushotham et al. [11] showed that the open group had more postoperative pain (mean VAS score: 7.48±0.42) than the laparoscopic group (mean VAS score: 3.05±0.01). This discrepancy was statistically significant, with a p-value of 0.047. In their study, Thota et al. [19] also found a decrease in postoperative discomfort in patients who underwent laparoscopic surgery. Our investigation revealed a significant variation in the incidence of postoperative seroma formation among various management groups (p=0.025). The incidence of surgical site infection was highest in cases that underwent open anatomical repair (41.7%), followed by RS repair (31.3%).

Purushotham et al. [11] discovered that the open group had a significantly higher incidence of postoperative wound infections (9.5% versus 0%) and recurrence (4.8% versus 0%) than the laparoscopic group in their study. Korukonda et al. [12] reported comparable findings regarding seroma occurrence, with an incidence of 3% in the open group and 0% in the laparoscopic group. In a study by Mohamed et al. [18], the infection rate was 5% in the open group and 1% in the laparoscopic group.

Olmi et al. [20] found that the prevalence of postoperative superficial SSI was higher in open patients than in closed patients (30% versus 10.7%; p<0.01) in their study. Liang et al. [21] also discovered that laparoscopic surgery was significantly associated with a lower incidence of surgical site infections (7.6% versus 34.1%; p<0.01). Lomanto et al. [17] also observed a lower incidence of complications (24%) and recurrences (2%) in individuals who underwent laparoscopic surgery compared to those who underwent open repair (30% and 10%, respectively). Furthermore, utilization of SSI bundles and strict adherence to institutional antibiotic prophylaxis policies can prove as useful methods to reduce postoperative SSI rates. The mean postoperative hospital stay of the laparoscopic group was significantly shorter than that of the open hernia group (1.15 versus 4.55 days; p=0.002).

Our study had a decent sample size, considering the limited duration of the study. Also, the inclusion/exclusion criteria and statistical methods employed were appropriate. Our research has attempted to deal with a clinically relevant topic, affecting people all over the world.

A small sample size, a lack of bias prevention methods, and a brief follow-up period are among the few deficiencies of the study. However, we are committed to expanding our research by incorporating a broader study cohort, which will yield more comprehensive data that will contribute to the ever-growing body of knowledge regarding abdominal wall hernias.

## Conclusions

The current study aimed to emphasize the diverse risk factors linked to the incidence of abdominal wall hernia and the numerous management approaches utilized for its treatment. A comprehensive understanding of these risk factors is essential since it enables surgeons to be diligent in both identifying them and preventing hernia recurrence post-surgical treatment, given their persistent nature. Our study demonstrated comorbidities, obesity, smoking, and prior surgery as significant risk factors for the occurrence of ventral hernia. Among the open and laparoscopic surgical procedures, laparoscopic methods had the upper hand in terms of shorter operative duration and lower incidence of postoperative pain, seroma, and early recurrence. Furthermore, the utilization of standard institutional protocols and SSI bundles can help in lowering SSI rates.

Hernia surgery constitutes the bread and butter of surgeons around the world. Therefore, it is crucial to meticulously choose the surgical approach (open or laparoscopic) depending on the patient's overall health and clinical symptoms in order to achieve the desired outcomes and minimize complications. Also, surgeon preference, availability of surgical expertise, and resources should also be taken into consideration in the choice of surgery, thereby ensuring optimum outcomes.
